# Diagnostic value of urinary luteinizing hormone levels in the monitoring of precocious puberty treatment

**DOI:** 10.20945/2359-3997000000212

**Published:** 2020-03-18

**Authors:** Özge Yüce, Aysun Bideci, Nurullah Çelik, Orhun Çamurdan, Peyami Cinaz

**Affiliations:** 1 Yildirim Beyazit University Faculty of Medicine Yenimahalle Training and Research Hospital Ankara Turkey Yildirim Beyazit University Faculty of Medicine, Yenimahalle Training and Research Hospital, Department of Pediatric Endocrinology, Ankara, Turkey; 2 Gazi University Faculty of Medicine Department of Pediatric Endocrinology Ankara Turkey Gazi University, Faculty of Medicine, Department of Pediatric Endocrinology, Ankara, Turkey; 3 Cumhuriyet University Faculty of Medicine Department of Pediatric Endocrinology Turkey Cumhuriyet University, Faculty of Medicine, Department of Pediatric Endocrinology, Turkey

**Keywords:** Monitoring, precocious puberty, urinary gonadotropin, GnRH analogues

## Abstract

**Objective:**

To determine whether first-voided urinary LH (FV-ULH) – level measurement can adequately assess pubertal suppression as much as standard tests can.

**Subjects and methods:**

The study group included patients with central precocious puberty and rapidly progressing early puberty who received up to 3 – 4 doses of GnRHa therapy monthly and did not have adequate hormonal suppression after GnRH stimulation (90-minute LH level > 4 IU/L). Design: All of the participants underwent an LHRH test just after admission to the study. According to the stimulated peak LH levels, the patients were divided into 2 groups and followed until the end of the first year of treatment. The concordance between FV-ULH and stimulated LH levels was assessed.

**Results:**

The FV-ULH levels in patients with inadequate hormonal suppression were significantly high compared to patients with adequate hormonal suppression. FV-ULH levels were very strongly correlated with stimulated LH levels (r = 0.91). Its correlation with basal LH levels was significant (r = 0.65). However, this positive correlation was modestly weakened after the first year of treatment. The cutoff value for FV-ULH of 1.01 mIU/mL had the highest sensitivity (92.3%) and specificity (100%).

**Conclusion:**

FV-ULH levels, using more reliable and sensitive assay methods, can be used to monitor the adequacy of GnRHa therapy.

## INTRODUCTION

The standard recommended treatment for central precocious puberty (CPP) is the depot form of gonadotropin-releasing hormone analogues (depot GnRHa) (1). Monitoring of treatment response should include assessment of Tanner stage and growth velocity every 3 to 6 months, along with periodic assessment of bone age advancement (2). However, these parameters are not always sufficient to predict the degree of gonadotropin suppression, particularly during initial treatment. In particular, the examiners had significant variability in their bone age interpretation (2). Therefore, stimulated luteinizing hormone (LH) levels following intravenous administration of luteinizing hormone-releasing hormone (LHRH) are accepted as the mainstay of treatment monitoring (2-4). However, this test requires venipuctures and multiple serum samples for LH, which is time-consuming, costly, and a tedious process for children. Recently, it has been shown that instead of using an LHRH test to evaluate treatment efficacy, one may use the LH level following the injection of depot GnRHa, which is considered more practical and reliable. Nevertheless, there is no consensus on when exactly the LH level should be measured following the administration of depot GnRHa. Another matter is what the cutoff level of LH should be in defining adequate hormonal suppression during the treatment period. Therefore, several studies have been done to determine the laboratory criteria in monitoring GnRHa treatment and to simplify the test.

Urinary gonadotropin measurement is one potential alternative approach for assessing the hormonal suppression under GnRHa treatment. However, the use of urinary gonadotropin measurement should be checked, primarily due to concerns about sensitivity of the assay. Additionally, consistency with serum and urinary gonadotropins levels should be demonstrated. Previous studies have suggested that urinary gonadotropin excretion not only reflects an integrated gonadotropin secretion (5,6) but is also correlated with the physical signs of pubertal development (7,8). Several recent studies support this, and current data on this subject is also promising (9,10).

In this prospective study, our primary objectives were to test the reliability of first-voided urinary LH (ULH)–level measurement and to determine whether it can predict pubertal suppression as much as standard tests can, such as the LHRH test or GnRHa test.

## SUBJECTS AND METHODS

### Study patients

The study group was selected from patients with CPP and rapidly progressing early puberty who had received up to 3-4 doses of monthly GnRHa therapy and did not have adequate hormonal suppression after GnRH stimulation (90-minute LH level > 4 IU/L) (11).

The pretreatment assessment included determination of height, weight, pubertal stage, bone age (BA), and target and predicted final height. Pubertal stage was evaluated by clinical examination according to Marshall and Tanner (12). Additionally, BA was evaluated according to the method developed by Greulich and Pyle (13). The target height was also calculated, using the conventional formula [TH: (mother’s height + father’s height)/2 + 6.5 (boys) and – 6.5 (girls)]. Predicted final height (PFH) was calculated using average Bayley and Pinneau tables (14). The patients showed no an evidence of an organic central nervous system disorder or of adrenal or gonadal disease.

The clinical criteria for a diagnosis of CPP and rapidly progressing early puberty were: (i) onset of pubertal signs before the age of 8 years or menarche before 9 years old, (ii) onset of pubertal signs at age 8-9 years but pubertal stage progression from one stage to the next in <6 months, (iii) increase in height velocity (≥ 6 cm/year), (iv) BA advance greater than 2 years above the chronological age (CA), (v) predicted adult height falling below 2 SD (about 10 cm) of the target height and acceleration of somatic development (15). Basal LH ≥ 0.3 mIU/mL, stimulated peak LH > 5 mIU/mL, and LH/follicle stimulating hormone (FSH) peak ratio ≥ 0.66 after LHRH administration were accepted as the laboratory criteria for CPP (15).

All of the patients were treated with either leuprolide acetate or triptorelin acetate, administered intramuscularly at a starting dosage of 3.75 mg every 28 days. Doses were incrementally adjusted by 3.75 mg, if necessary, based on the results of the LHRH test and pubertal progression.

Ethics committee approval was obtained from the Institutional Ethics Committee of Gazi University Faculty of Medicine. Written informed consent was obtained from patients and their parents.

### Study design

The treatment’s effectiveness was evaluated every 3-4 months, based on a set of clinical and hormonal data, such as regression or arrest pubertal progression, decrease in growth velocity, reduction of bone age advancement, and improvement in final height prediction (16).

Hormonal suppression was assessed using an LHRH test and after GnRHa stimulation. A cutoff value of 2 mIU/mL or less for peak stimulated LH during the LHRH test, and 90-minute LH level at < 4 mIU/mL after the GnRHa injection was deemed to show adequate hormonal suppression (11,17). Basal concentration of LH was also measured during these tests.

The LHRH test was performed twice. First, it was used to classify the patients based on their peak-stimulated LH levels. Group 1 consisted of patients who had adequate hormonal suppression (peak stimulated LH ≤ 2 mIU/mL), while the remaining patients were group 2. Doses were only adjusted in group 2 patients with pubertal progression.

**Figure 1:**
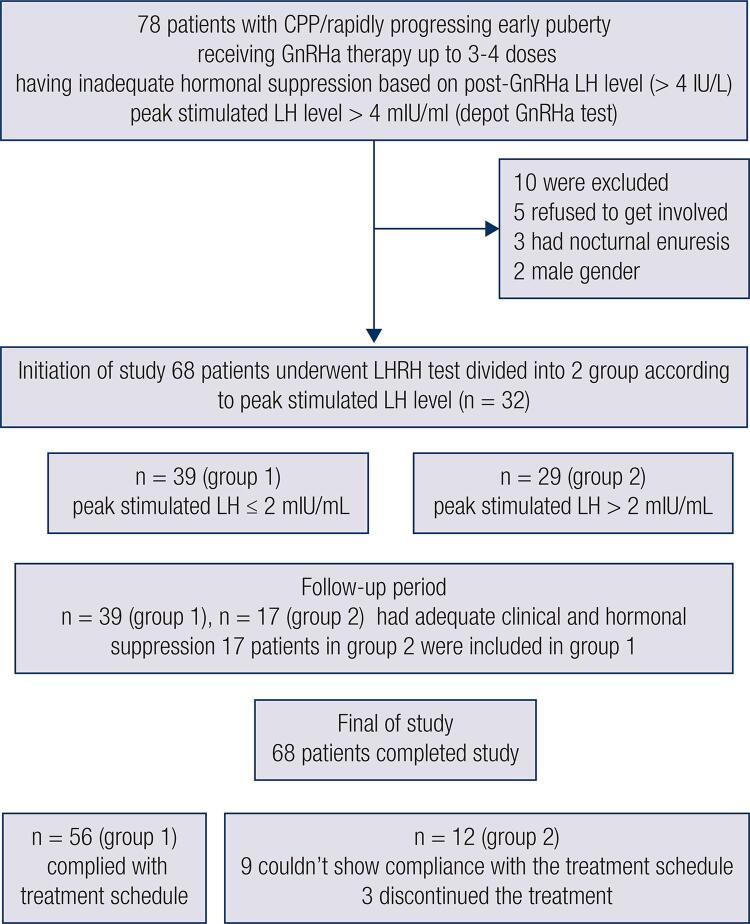
Patients dispositions flow chart

GnRHa-stimulated LH levels were obtained from all patients in the first control after grouping. The second LHRH test was performed in the first year of the study (Figure 1).

Patients were excluded if they had a history of nocturnal enuresis, any urinary tract disease, diabetes, or metabolic or neurologic disease.

### Urinary samples

The first-voided urine samples were taken from the patients. For reliable evaluation of ULH, all of patients were instructed to empty their bladder before bedtime and to refrain from voiding until the next morning. Both serum and urinary LH levels were assayed on the same day of the clinic visit. The urine samples were not stored frozen.

### Assays

Serum and urinary LH levels were measured using electrochemiluminescence assay (ECLIA) (Cobas 6000, Roche Diagnostics, Mannheim, Germany). The minimum detectable LH level was 0.01 IU/L. The intra-and interassay coefficients were 3.1% and 4.1%, respectively.

### Statistical analysis

All of the statistical calculations were performed using SPSS version 20 (SPSS, Inc., Chicago). Descriptive statistics were computed as means ± standard deviation (SD). Parameters with normal distribution were analyzed with t-tests, and parameters with nonnormal distributions were analyzed with Mann-Whitney U-tests. The linear relationships between variables were evaluated using Spearman’s correlation tests. A *P* value < 0.05 was considered statistically significant. The diagnostic value of FV-ULH was evaluated using receiver-operating characteristics (ROC) analysis.

## RESULTS

Although 78 patients were included in this study, 5 of them were excluded because they refused to become involved in the study, 3 were excluded because they had nocturnal enuresis, and 2 boys were excluded from the study in order to prevent gender factors in the assessment.

Finally, this study started with 68 female patients, of whom 56 patients complied with the treatment schedule and appointments. Nine of remaining the 12 patients did not comply with the treatment schedule because they could not obtain the drug. Due to personal reasons unrelated to the treatment, 3 patients discontinued the treatment (Figure 1).

All of the patients demonstrated similar baseline demographics and stages of puberty, as shown by the Tanner staging. Chronological age of pubertal onset in patients was at 8.22 ± 1 years old. While 32 patients (47%) were at Tanner stage 2, 34 (50%) were at stage 3. Only 2 patients had stage 4 pubertal development, of whom 1 presented menarche. Twenty patients (29.4%) had a diagnosis of rapidly progressing early puberty. All of the patients had been treated for a maximum of 1 year from the initiation of the study (mean doses of treatment 12.2 ± 1.8). The patients’ pretreatment clinical characteristics are summarized in Table 1.

**Table 1:** Baseline clinical charactesristics of 68 patients with CPP/rapidly progressing early puberty

	Group 1 (n: 36)	Group 2 (n: 32)	Overall
Chronologic age at diagnosis (year)	8.04 ± 0.75	8.44 ± 0.64	8.22 ± 1.32
Height age at diagnosis (year)	9.17 ± 1.01	9.55 ± 0.9	9.35 ± 1.0
Bone age at diagnosis (year)	9.85 ± 1.05	9.92 ± 1.05	9.9 ± 1.04
Height at diagnosis (cm)	132 ± 5,75	134.6 ± 5,7	133.2 ± 5.8
Weight at diagnosis (kg)	32.5 ± 5.6	35 ± 6.9	33.6 ± 6.3
Target height (cm)	159.8 ± 3.1	160.2 ± 6.5	160 ± 4.8
Predicted height (cm)	153.8 ± 4.6	156 ± 3.4	154.9 ± 4
Tanner stages at diagnosis			n (%)
Stage II			32 (47%)
Stage III			34 (50%)
Stage IV (presented with menarche)			2
Mean basal LH (mIU/mL)	0.43 ± 0.47	0.64 ± 0.54	0.53 ± 0.41
Peak stimulated LH level at diagnostic LHRH (mIU/mL)	8.2 ± 0.65	8.5 ± 1.9	8.35 ± 1.23

CPP: central precocious puberty; SD: standard deviation; LH: luteinizing hormone; LHRH: luteinizing hormone releasing hormone.

### Initiation of the study

Group 1 comprised 39 patients. The post-GnRHa LH levels of these patients was 4.13 ± 0.03 mIU/mL (considered inadequate suppression), but all had successfully suppressed peak LH values (≤ 2 mIU/mL) in response to the LHRH. Pubertal regression was also provided to all these patients.

Group 2 was composed of 29 patients. Pubertal staging remained unchanged in 24 of the patients, advanced by one Tanner stage in 3 patients, and regressed in 2 patients, but none of the patients achieved hormonal suppression in response to the LHRH (2.83 ± 0.47, *P* < 0.001) or to the post-GnRHa (4.67 ± 0.16, *P* < 0.001). We increased the dosage by 7.5 mg/mo in the 3 patients who showed pubertal progression.

Group 2 had significantly higher FV-ULH, basal LH, and peak-stimulated LH levels than group 1 did (Table 2). FV-ULH was significantly correlated with the basal LH levels (*r* = 0.65, *P* < 0.001) and had a very strong correlation with peak stimulated LH levels (*r* = 0.91, *P* < 0.001).

**Table 2:** Serum and FV-ULH levels at the first 3 months of treatment. Data are presented as mean ± SD with range of values in parentheses and the mean value with min-max values were calculated for FV-ULH

	Group 1 (n = 39)	Group 2 (n = 29)	p value
Basal LH	0.11 ± 0.07	0.4 ± 0.27	p < 0.001
Peak LH (mIU/mL) to LHRH	1.64 ± 0.25	2.83 ± 0,47	p < 0.001
90 min.LH (mIU/mL) to depot GnRH	4.13 ± 0.03	4.67 ± 0.16	p < 0.001
FV-ULH (mIU/mL)	0.35 ± 0.15	1.3 ± 0.079	p < 0.001
Median FV-ULH (min-max)	0.31 (0.12-0.71)	1.31 (1.17-1.4)	

SD: standard deviation; LH: luteinizing hormone; LHRH: luteinizing hormone releasing hormone; FV-ULH: first voiding urinary luteinizing hormone.

### Follow-up period of the study

After the sixth or seventh dose of GnRHa therapy, all of group 1 (39 patients) and only 17 of the 29 patients in group 2 were examined clinically and received hormonal evaluation. Fourteen of the patients in group 2 showed pubertal regression; however, pubertal staging remained unchanged in 3 patients. All 56 patients (39 patients in group 1, the remaining in group 2) demonstrated hormonal suppression (90-minute LH levels < 4 mIU/mL to the GnRHa). Afterward, 17 patients in group 2 were included in group 1 and followed up.

The groups did not show statistical differences in basal or stimulated LH levels. However, FV-ULH levels were significantly different between from Group 1 (0.26 ± 0.15 mIU/mL) and group 2 (0.68 ± 0.14 mIU/mL) (*P* < 0.05).

### Final stage of the study

Of the patients, 68 could be evaluated after 1 year of treatment. Pubertal regression and hormonal suppression were maintained in 56 patients, all of whom were in group 1. Twelve patients remained in group 2, including 9 patients who did not show treatment compliance. Of these patients, 7 showed no change in pubertal staging, while 2 patients showed pubertal progression. The other 3 patients in group 2 who discontinued the treatment showed pubertal progression. All 12 patients continued to exhibit pubertal hormone levels. Both basal and peak-stimulated LH levels in response to the LHRH were significantly higher among group 2 than in group 1. Group 2’s FV-ULH levels were 1.4 ± 0.4 mIU/mL, which was also significantly high compared to group 1 patients (Table 3). Their FV-ULH levels were measured as 0.31 ± 0.24 mIU/mL. The correlation between FV-ULH and basal LH levels was weakly positive (r = 0.4).

**Table 3:** Serum and FV-ULH levels after 1-year of treatment. Data are presented as mean ± SD with range of values in parentheses

	Group 1 (n = 56)	Group 2 (n = 12)	p value
Basal LH	0.15 ± 0.08	0.5 ± 0.16	p < 0.001
Peak-stimulated LH (mIU/ml) to LHRH	1.42 ± 0.21	3.53 ± 0.4	p < 0.001
FV-ULH (mIU/mL)	0.31 ± 0.24	1.4 ± 0.4	p < 0.001

SD: standard deviation; LH: luteinizing hormone; LHRH: luteinizing hormone releasing hormone; FV-ULH: first voiding urinary luteinizing hormone.

**Figure 2:**
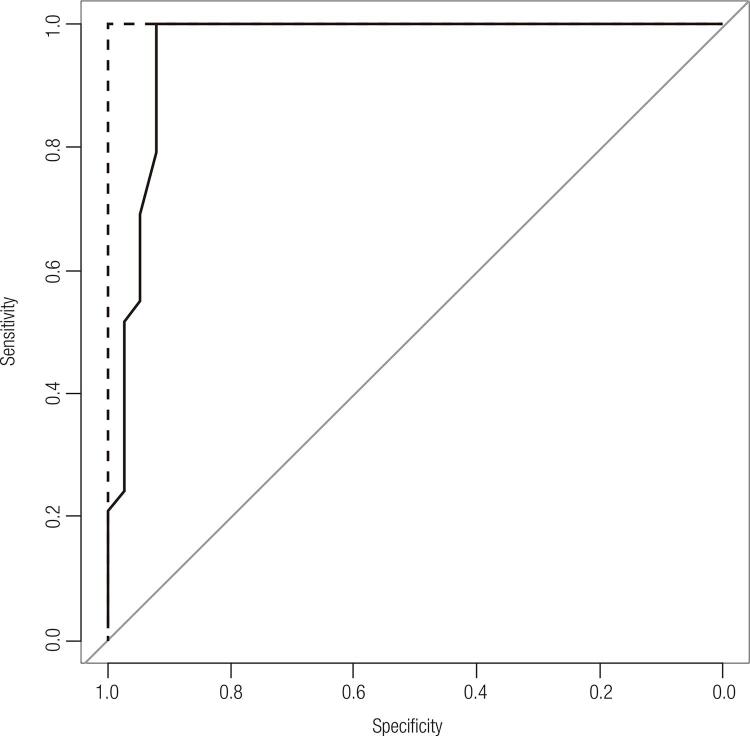
Receiver-operating characteristics curve of first-voided urinary LH (FV-ULH) levels. The solid line represents the cutoff level of FV-ULH at the initiation of the study. The dotted line represents the cutoff level of FV-ULH at the final of the study.

The ROC analysis demonstrated that the sensitivity (92.3%; CI, 0.91-1, 79.1%-98.4%) and specificity (100%; CI, 88.1%-100%) of FV-ULH were the highest when the cutoff FV-ULH value at the study’s initiation was > 1.01 mIU/mL. Its sensitivity and specificity at the end of the study were both 100%. The sensitivity and specificity of different cutoffs are shown in Figure 2.

## DISCUSSION

In this prospective study, we showed that FV-ULH can be used to assess pubertal suppression throughout GnRHa treatment in children with CPP. We found that FV-ULH levels among patients with adequate and inadequate hormonal suppression in response to GnRHa treatment were significantly different from each other. We found that FV-ULH levels were at least as sensitive as traditional LHRH stimulation and/or GnRHa tests in monitoring treatment. We also set a cutoff value with high sensitivity and specificity.

Urinary gonadotropin measurements have been used to evaluate gonadotropin secretion in children since the 1960s; however, the first studies were fairly in agreement that gonadotrophins were not detectable in the urine with current measurement techniques and therefore suggested that more sensitive methods should be developed to assay urinary gonadotropins (18).

Over time, the standards of practice for urinary gonadotropins were established, and sensitive assay methods were also developed. Urinary gonadotropins have also been studied in different age and pubertal groups as well as in patients with puberty disorders (19-21). These studies have suggested that noninvasive urinary gonadotropin measurement could be a viable alternative to measuring serum levels. Yet today, sports physicians measure urinary gonadotropins to determine doping in athletes (22,23), but it is hardly used in endocrinology practice.

Several recent studies measuring gonadotropin levels in the urine of preterm or full-term newborn infants have been reported. In these studies, the postnatal activation of the hypothalamic–pituitary–gonadal (HPG) axis was evaluated by using serial urine samples. The results of the urinary gonadotropin measurements were found to accurately describe the postnatal activation of the HPG axis, as compared to serum samples (24-27). Similarly, Demir and cols. (28) checked the reliability of urinary gonadotropin measurement and its availability in children with pubertal disorders. They showed that FV-ULH determination could be used as an alternative to the GnRH test in assessing HPG axis activity. Another study conducted by Zung and cols. was aimed at evaluating the diagnostic value of FV-ULH compared with that of GnRH-stimulated gonadotropins in predicting pubertal course and differentiating slowly progressive (SP) from rapidly progressive PP (RP-PP) in girls. Finally, they suggested that FV-ULH may help in determining the progression rate of puberty and thus supported the usage of urinary gonadotropin measurement (9). Just like Witchel and cols. did (29), in another study, they assessed whether the FV-ULH measurement was an alternative to a LHRH stimulation test for monitoring treatment for CPP (10). No correlation was shown between urinary and serum-stimulated LH levels. Therefore, in both studies, the results showed that urinary gonadotropin measurement could not replace the LHRH stimulation test for monitoring GnRHA therapy. However, neither study could make any predictions about the reliability of ULH since these studies were carried out in small groups of patients with CPP. Most importantly, the number of unsuppressed patients was not enough to determine whether ULH is useful for predicting treatment response.

Because our study included patients without hormonal suppression in response to treatment, it provides better knowledge about the usage of ULH. It is possible that the FV-ULH levels in our study, which reflect nocturnal excretion of LH (20), included a few episodes of escape from the HPG suppression that were not otherwise recognized by the LHRH stimulation test. Therefore, our study results may have high sensitivity and specificity. However, this can lead to inappropriate interventions by reflecting episodes that almost escaped HPG suppression, especially during the first months of treatment. For this reason, our results should be evaluated with clinical findings and confirmed with recurrent measures. This may be considered a possible limitation of the study. However, the main limitation of this study was the small number of unsuppressed patients, especially in the final evaluation of the study. Most of these unsuppressed patients did not show any compliance with the treatment protocol; therefore, their hormone levels remained within pubertal levels. As a result, significant differences in the urinary LH levels were observed between the groups, as well as high sensitivity and specificity. Additionally, we want to account for the potential limitation of the urinary gonadotropin measurements of obtaining LH from urine, which is a problem if the urinary samples are stored frozen; much of the LH can be degraded during long-term storage of urine at –20 °C (30). We prevented this possibility by measuring urine LH levels immediately.

As a result, measuring urinary gonadotropins with recent assay methods is highly reliable due to its sensitivity. The use of urinary samples for hormonal evaluation has become a popular yet controversial issue. Through our study, we can suggest that measuring ULH levels can provide accurate information about hormonal suppression, at least as accurately as serum LH levels can, without the burden of frequent blood sampling. Additionally, our results encouraged us to use urinary gonadotropin measurement in diagnosing and following up other pubertal disorders. Certainly, further comprehensive studies with larger groups will be required to clarify the utility of urinary gonadotropin measurement for identifying pubertal disorders.
